# Adult-Onset Craniotubular Dysplasia, Ikegawa Type: A Report of a Case From Bahrain With a Novel Compound Heterozygous TMEM53 Mutation and Literature Review

**DOI:** 10.7759/cureus.107793

**Published:** 2026-04-27

**Authors:** Bayan M Barni, Ameera M Barni, Ebtihal Alyusuf, Amna Alawadhi, Sarah Al Mail

**Affiliations:** 1 Internal Medicine, Salmaniya Medical Complex, Manama, BHR; 2 Endocrinology and Diabetes, Bahria University Medical and Dental College, Manama, BHR; 3 Genetics, Salmaniya Medical Complex, Manama, BHR; 4 Radiology, Salmaniya Medical Complex, Manama, BHR

**Keywords:** bahrain, ctdi, osteoprosis, stenosis, tmem53

## Abstract

Craniotubular dysplasia, Ikegawa type (CTDI), is an ultra-rare sclerosing bone disorder associated with pathogenic mutations in the TMEM53 gene. The condition disrupts the negative regulation of ossification, leading to progressive skeletal abnormalities, short stature, and early-onset visual impairment secondary to optic nerve compression. Since its initial description in 2021, only a limited number of cases have been reported, nearly all presenting in childhood. Adult-onset disease remains exceptionally rare and poorly characterized.

In this report, we describe a 38-year-old Bahraini gentleman with a 10-year history of progressive left-sided numbness, severe musculoskeletal and back pain, gait disturbance, and unilateral blindness. His presentation was insidious with a gradual neurological decline and predominant spinal manifestations attributable to spinal pathology, which is an atypical presentation compared to previously reported cases that primarily presented with craniofacial involvement. Magnetic resonance imaging (MRI) demonstrated long-segment cervical canal stenosis with spinal cord compression and edema, Schmorl’s nodes, thoracic disc bulges, and lumbar canal stenosis. Despite undergoing two spinal decompression surgeries, his neurological symptoms persisted. Comprehensive genetic evaluation, including whole-exome sequencing, identified two novel rare missense variants in TMEM53 (p.Ala145Pro and p.Pro211Thr) in a compound heterozygous state, establishing the diagnosis of craniotubular dysplasia, Ikegawa type (CTDI), and expanding its allelic spectrum.

This case broadens both the phenotypic and genotypic spectrum of CTDI. It provides the most detailed spinal imaging description of this disorder to date and highlights an unusual adult-onset presentation with predominant spinal involvement and unilateral blindness.

## Introduction

Craniotubular dysplasia, Ikegawa type (CTDI) (OMIM #619727), is a rare autosomal recessive skeletal disorder caused by pathogenic variants in the TMEM53 gene [1,2].

The TMEM53 (OMIM 619722) gene encodes nuclear envelope transmembrane protein 53 (NET53), which plays a critical role in the negative regulation of bone formation through the inhibition of the bone morphogenetic protein (BMP) signaling pathway [3,4]. The BMP pathway begins with the binding of BMP2 or BMP4 ligands to their respective receptors, activating an intracellular signaling cascade involving the Suppressor of Mothers against Decapentaplegic 1 (SMAD1), SMAD5, and SMAD9. These phosphorylated SMADs form complexes with SMAD4, which then translocate from the cytoplasm into the nucleus and activate transcription of target genes that promote bone formation (osteogenesis) [5,6].

The TMEM53 gene functions as a suppressor of this BMP pathway. Loss-of-function mutations or deficiency of TMEM53 leads to unchecked BMP signaling, resulting in excessive bone formation and increased bone density. This dysregulated ossification contributes to the skeletal abnormalities observed in CTDI [7,8]. CTDI is classified among the sclerosing bone disorders (SBD) that typically present with proportionate short stature or short limbs, often accompanied by macrocephaly, dolichocephaly, or a prominent forehead [9-11].

A hallmark feature is early-onset visual impairment that progresses to blindness secondary to optic nerve compression from progressive cranial hyperostosis [12,13]. Radiographically, CTDI is characterized by hyperostosis of the skull base and calvaria, occasionally with diploic thickening, along with mild platyspondyly [14]. The long tubular bones display metadiaphyseal undermodeling (under-constriction), whereas short tubular bones may show mild shortening and diaphyseal broadening [9,15].

Here, we report a Bahraini male patient found to harbor compound heterozygous variants in TMEM53, resulting in a diagnosis of CTDI. We also provide a review of the existing literature, highlighting the expanding phenotypic and genotypic spectrum of this ultra-rare disorder.

## Case presentation

This case pertains to a 38-year-old male patient with a known history of diabetes mellitus, obesity, short stature, psoriasis, and migraine. He was born to consanguineous parents and is one of 12 siblings. Notably, four of his siblings reported similar medical conditions, suggesting a possible inherited genetic disorder.

The patient was previously in his usual state of health until approximately 10 years prior to presentation, when he developed a progressive left-sided numbness involving the entire left hemibody, including the left eye and ear. On examination, the patient demonstrated left-sided sensory deficit, gait disturbance, musculoskeletal tenderness over the spine, and reduced functional mobility with features consistent with chronic cervical myelopathy. Given the persistence of back pain accompanied by sensory deficits, comprehensive spinal imaging was performed. The subsequent sections detail the most clinically significant radiological findings.

Computed tomography (CT) of the spine

**Figure 1:**
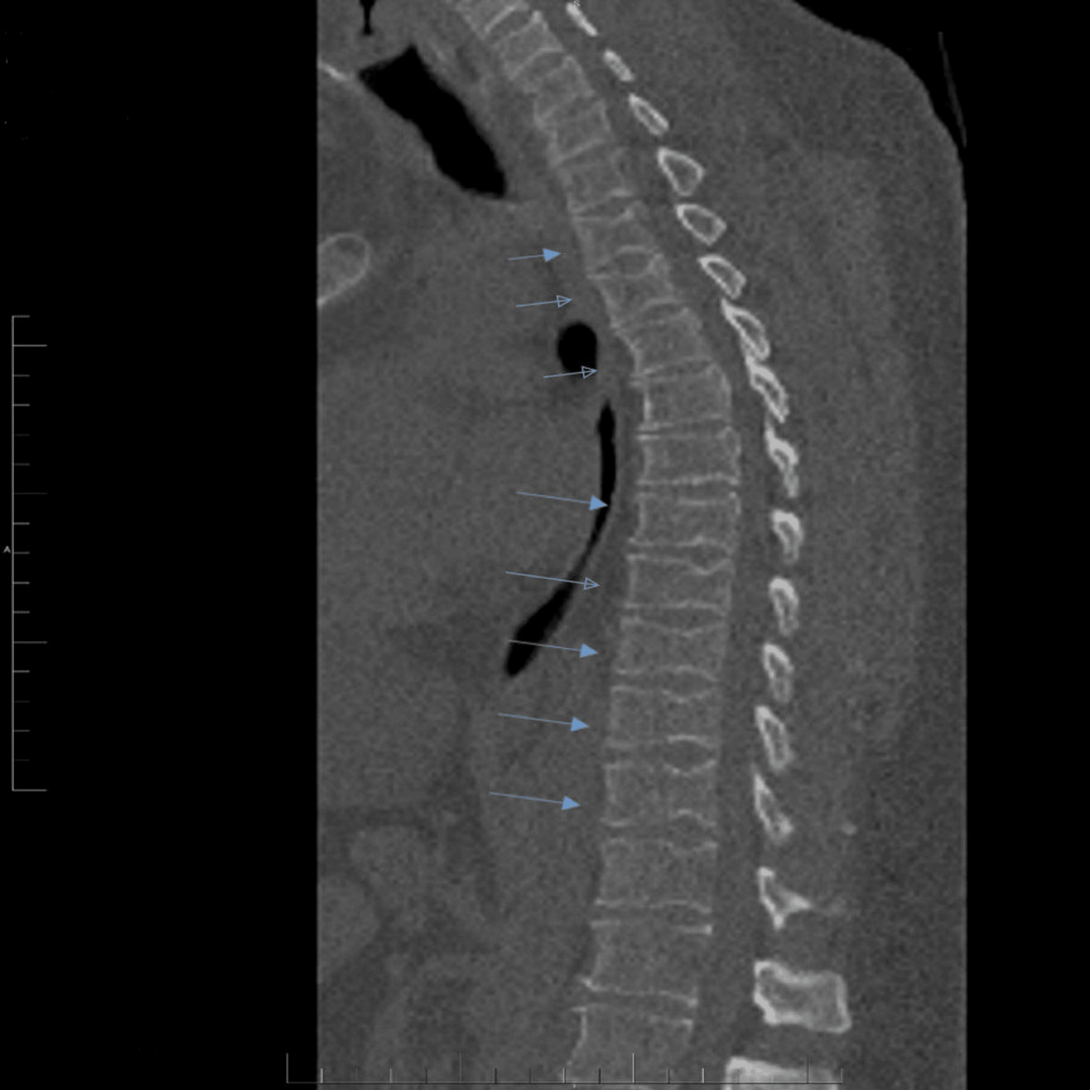
Sagittal non-contrast CT of the thoracic spine demonstrating platyspondyly (flattening of the vertebral bodies). The blue arrows indicate the affected vertebral bodies.

CT scan of the entire spine was performed on October 18, 2022. The study demonstrated straightening of the cervical and thoracic spine, likely secondary to paraspinal muscle spasm. Multiple H-shaped vertebrae were identified within the dorsal spine. Reversal of the normal cervical lordosis was noted, with preservation of the lumbosacral lordosis. Vertebral body heights and intervertebral disc spaces were preserved apart from C6-7 narrowing with no evidence of acute vertebral compression. No acute fractures were identified. Platyspondyly, characterized by flattened vertebral bodies, was observed throughout the axial skeleton (Figure 1).

Magnetic resonance imaging (MRI) of the spine

**Figure 2:**
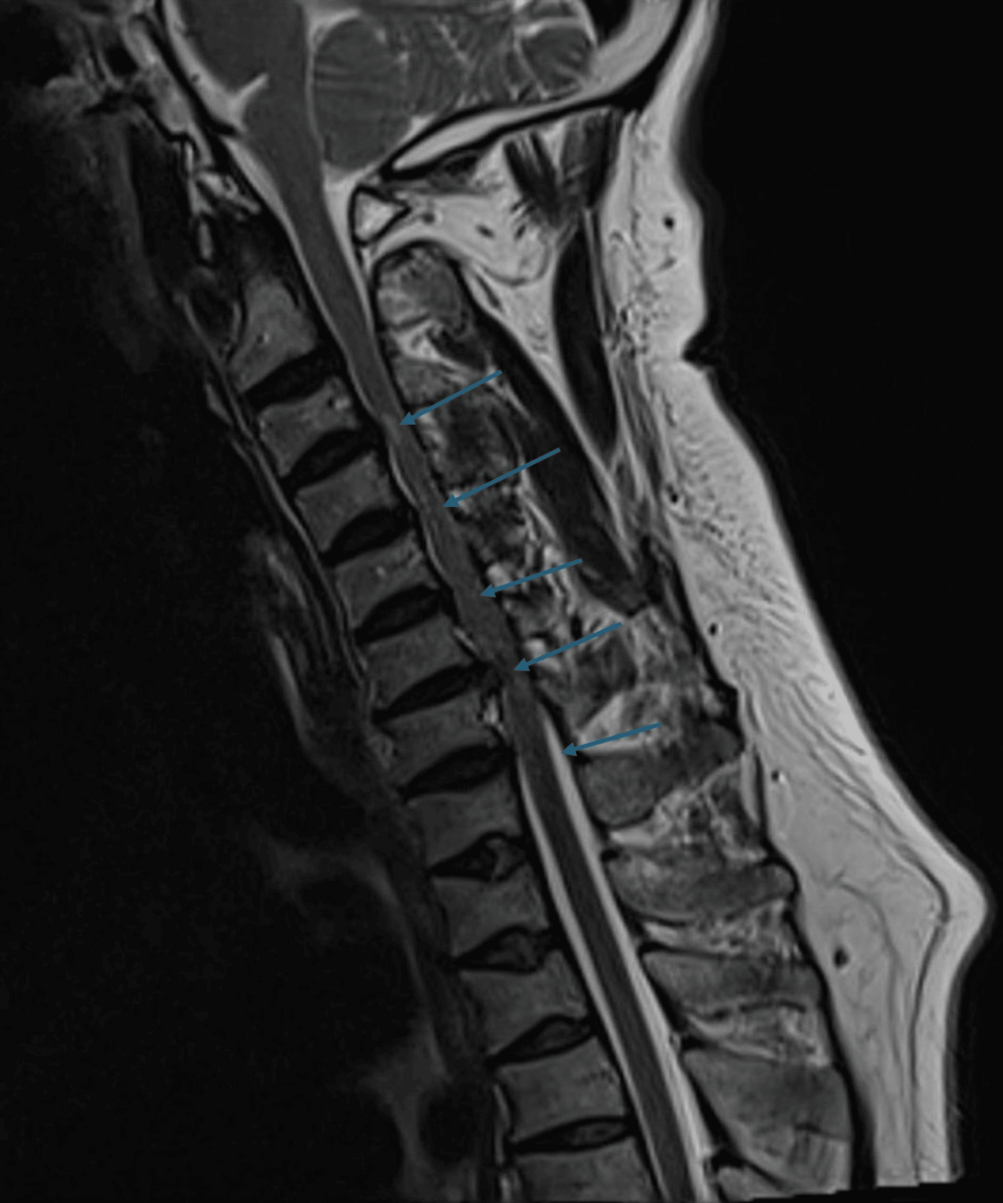
Sagittal T2-weighted MRI of the cervicothoracic spine demonstrating multilevel cervical central canal stenosis. The blue arrows indicate the sites of stenosis.

**Figure 3:**
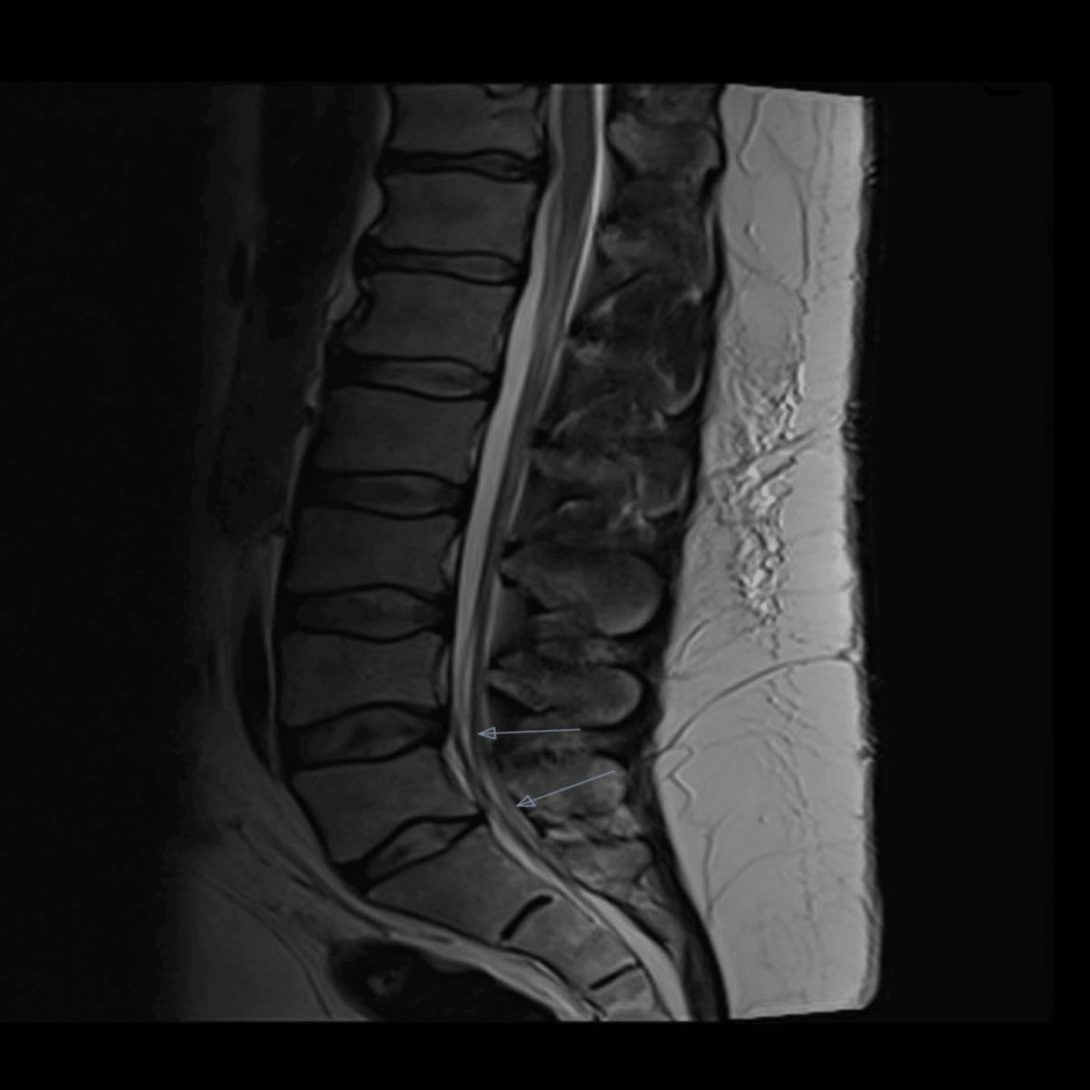
Sagittal T2-weighted MRI of the lumbosacral spine demonstrating spinal canal stenosis at the L4–L5 and L5–S1 levels. The blue arrows indicate the sites of stenosis.

Whole-spine MRI demonstrated diffuse fatty marrow conversion suggestive of osteopenia and multilevel degenerative changes. Long-segment cervical spinal canal stenosis extending from C3-C4 to C6-T1 was identified, associated with multilevel disc bulges from C3-C6. At C3-C4, a right-sided disc bulge caused foraminal narrowing with compression of the exiting nerve root and focal T2 cord hyperintensity consistent with focal myelopathy. More pronounced compression was observed at C5-C6 and C6-C7, with a large disc-osteophyte complex at C6-C7 producing severe canal stenosis, marked spinal cord compression, and cord edema. In the thoracic spine, multilevel Schmorl’s nodes were noted, with a mild disc bulge at T4-T5 indenting the anterior cord and a left-sided disc-osteophyte complex at T12-L1 compressing the left traversing nerve root. In the lumbosacral region, degenerative changes with central canal narrowing were observed at L4-L5 and L5-S1. A follow-up lumbar MRI performed in March 2024 showed no significant interval progression (Figures 2, 3).

Surgical intervention

Following the imaging findings, which revealed a large disc prolapse at C6-C7 causing spinal cord compression, an anterior cervical discectomy and fusion (ACDF) was performed. The affected cervical disc was removed via an anterior approach, and fusion was achieved using an interbody cage implant. An 8-mm cage was inserted between the C6 and C7 vertebral bodies to maintain disc height and facilitate fusion. The posterior longitudinal ligament (PLL) was also addressed during the procedure.

Despite undergoing ACDF for cervical cord decompression, his symptoms showed minimal improvement, and he continues to experience persistent musculoskeletal and back pain, limiting his ability to sit or stand for prolonged periods and significantly impairing his occupational functioning. He remains on analgesic therapy, including nonsteroidal anti-inflammatory drugs and topical preparations.

Progression of articular and ocular manifestations

Approximately four years prior to presentation, the patient began complaining of severe bone and joint pain, particularly involving the knees, which was exacerbated by exertion and climbing stairs. Subsequently, he developed difficulty with ambulation. A lower limb (LL) radiograph was performed, which demonstrated one of the classic features of the disease, namely, mild cortical thickening and widening of the bone shaft.

**Figure 4:**
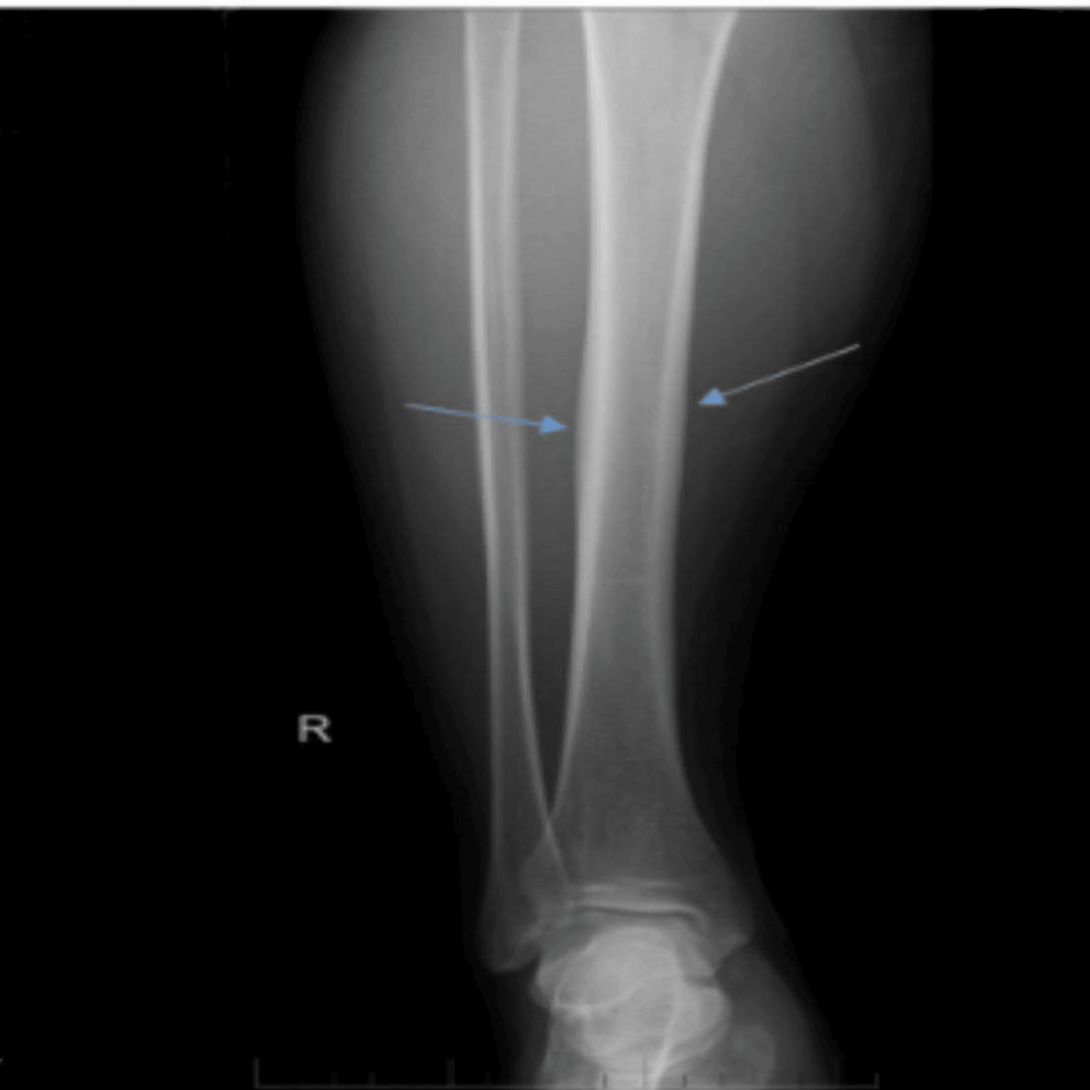
Anteroposterior (AP) radiograph of the right leg showing cortical widening and thickening of the diaphysis. The blue arrows indicate the affected areas.

For management, the patient was maintained on analgesics and was also prescribed liraglutide for a period of time to promote weight reduction, with the aim of alleviating joint pain (Figure 4).

**Figure 5:**
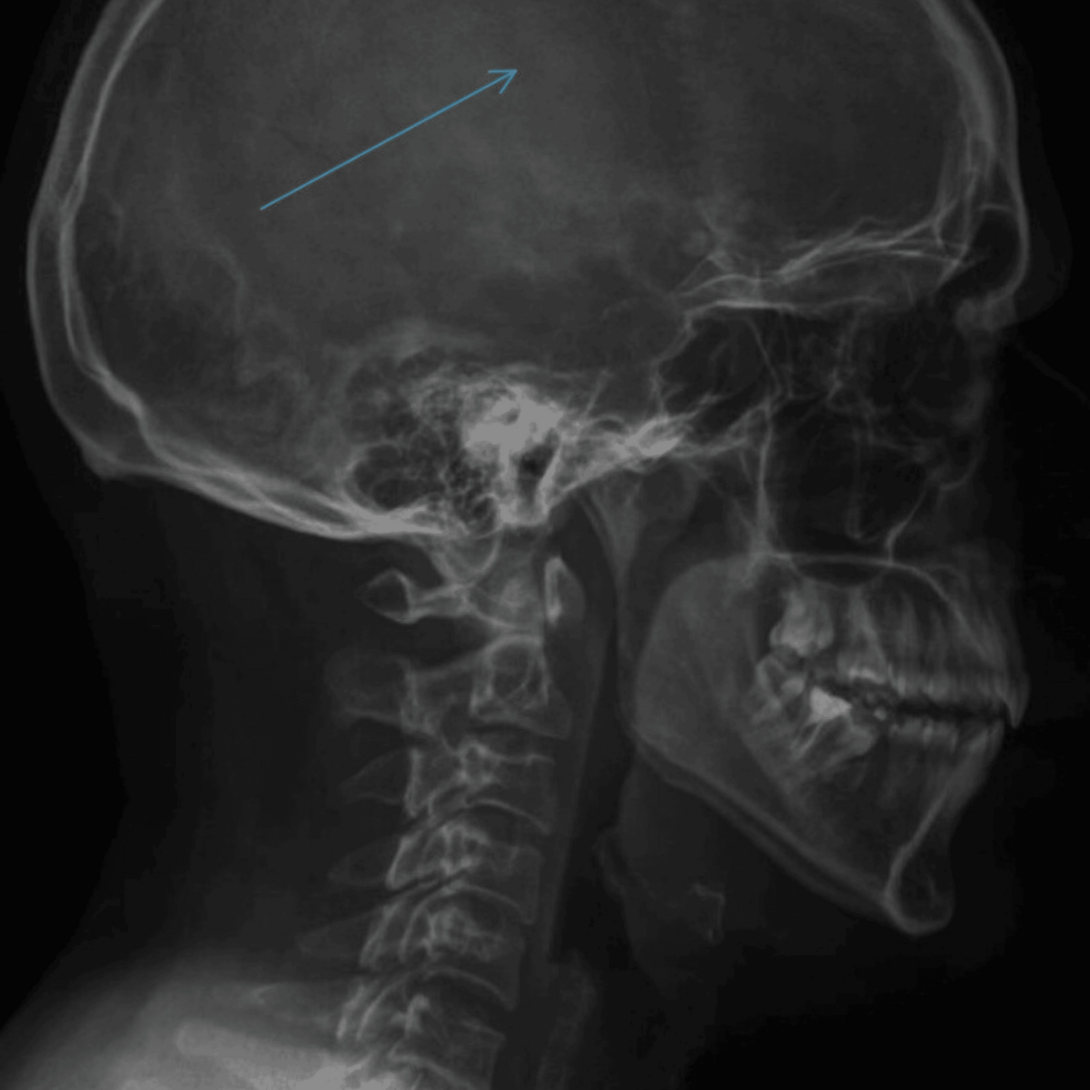
A Lateral skull radiograph demonstrates preserved calvarial contour and intact skull base.

**Figure 6:**
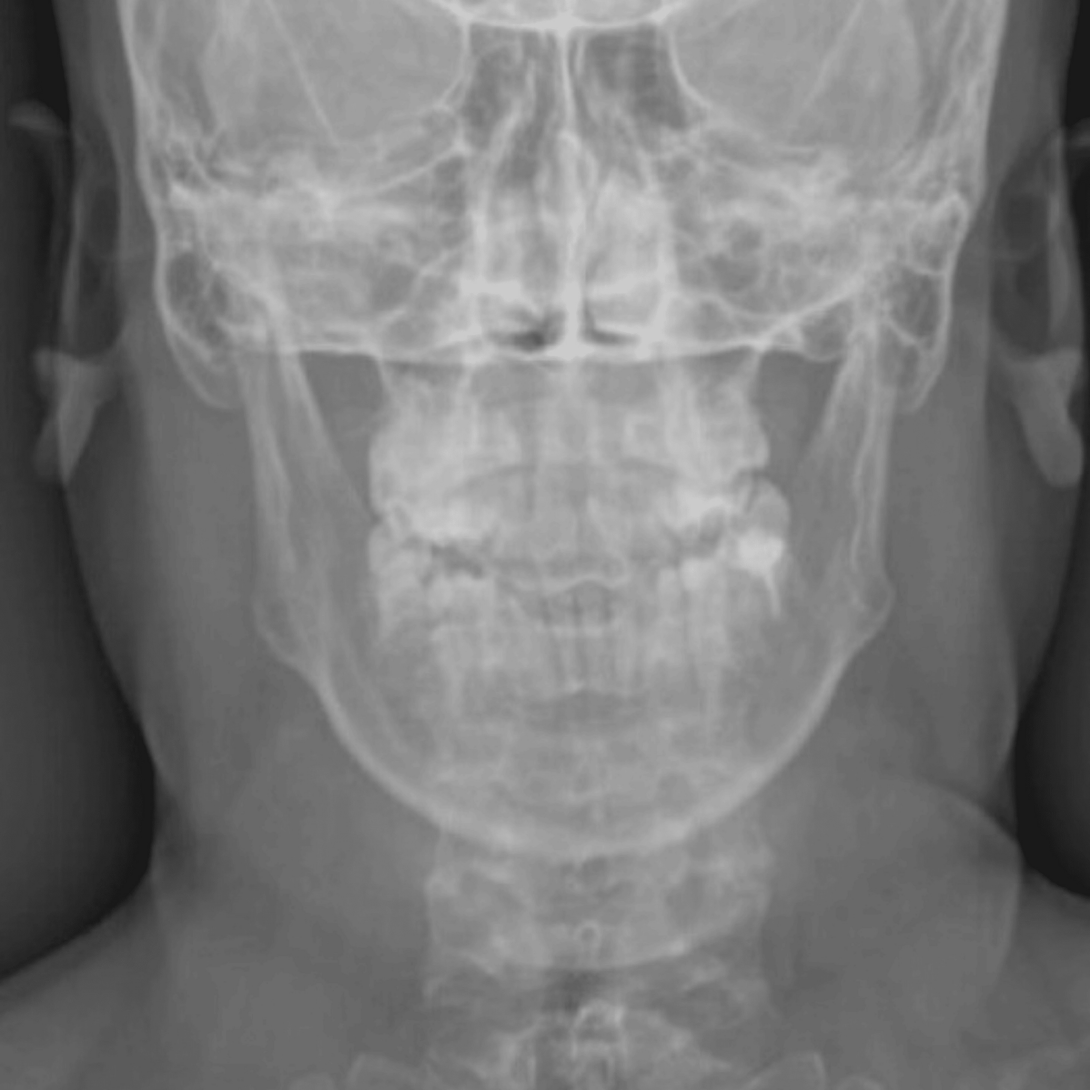
Frontal skull radiograph showing maintained cranial symmetry without features of sclerosing cranial dysplasia.

It is important to note that the patient also underwent a skull radiograph, which did not demonstrate definitive features of the disease. However, a CT scan is considered superior for a more accurate assessment of the skull base (Figures 5, 6).

In addition, the patient exhibited ophthalmologic problems, predominantly affecting the left eye. He developed progressive visual impairment in the left eye. Optic nerve compression was suspected as an underlying mechanism. Furthermore, corneal pathology consistent with keratoconus was also identified in the left eye, for which he underwent two corneal implant procedures; unfortunately, both were unsuccessful, ultimately resulting in complete loss of vision and left-eye blindness.

Osteoporotic involvement

The patient also had a history of osteoporosis and prior glucocorticoid exposure. On September 17, 2024, a bone mineral densitometry (DEXA) scan was performed, which demonstrated a bone mineral density below the expected range for age, consistent with generalized low bone mass and osteoporosis (Table 1).

**Table 1:** Bone mineral densitometry (BMD) results AP: Anteroposterior.

Site	Total BMD (g/cm²)	Z-score	Neck/Distal BMD (g/cm²)	Z-score
AP Spine (L1–L4)	0.955	-2.8	–	–
Left Femur	0.843	-2.0	0.794 (neck)	-2.3
Right Femur	0.862	-1.9	0.812 (neck)	-2.2
Left Radius	0.729	-0.3	0.958 (distal third)	-0.3

Other laboratory investigations demonstrated values within normal limits, including serum calcium (2.24 mmol/L), parathyroid hormone (PTH) (7.3), 25-hydroxyvitamin D (50 nmol/L), phosphate (1.0 mmol/L), magnesium (0.82 mmol/L), and alkaline phosphatase (68 U/L). He was started on zoledronic acid as an antiresorptive, aiming to suppress bone remodeling and formation with regular follow-up by orthopedic and metabolic bone disease clinics.

Genetic analysis

Methodology

To determine the underlying etiology of the patient’s manifestations and establish the diagnosis, whole-exome sequencing (WES) was performed on genomic DNA extracted from peripheral blood through an international laboratory. Coding regions and flanking intronic sequences (±10 bp) were enriched using hybridization capture methods targeting approximately 41 Mb of the exome. Sequencing was performed on an Illumina platform (CENTOGENE GmBH, Rostock, Germany) with a minimum coverage depth of 20× for over 99% of targeted regions. The analysis also included the mitochondrial genome. A comprehensive bioinformatics pipeline was applied for alignment to the GRCh37/hg19 reference genome, variant calling, annotation, and interpretation.

Variant prioritization focused on rare variants (minor allele frequency <1%) affecting protein-coding or canonical splice sites, and interpretation followed the American College of Medical Genetics and Genomics and the Association for Molecular Pathology (ACMG/AMP) and ClinGen guidelines. Analysis also included evaluation for copy number variants (CNVs), mitochondrial variants with heteroplasmy ≥15%, and selected uniparental disomy (UPD) regions.

Genetic Findings

No pathogenic or likely pathogenic variants clearly related to the patient’s phenotype were detected. However, two rare missense variants of uncertain significance (VUS) were identified in the TMEM53 gene: M_024587.3:c.433G>C, p.(Ala145Pro) and NM_024587.3:c.631C>A, p.(Pro211Thr).

Both variants are heterozygous, with deleterious predictions by multiple in silico tools (Sorting Intolerant From Tolerant (SIFT), PolyPhen-2, MutationTaster), and are absent or extremely rare in population databases (gnomAD allele frequencies: 0.000032 and not reported, respectively). Parental testing is necessary to determine whether these variants are in trans, as the disease associated with this gene follows an autosomal recessive inheritance pattern. Unfortunately, it could not be done as both parents passed away. Given the great phenotype-genotype correlation, we report this case as another compound heterozygous missense mutation for craniotubular dysplasia, Ikegawa type.

## Discussion

Craniotubular dysplasia represent a heterogenous group of sclerosing bone disorders characterized by abnormal bone modeling and sclerosis affecting the skull and tubular bones. These conditions encompass multiple subtypes with variable clinical and radiological manifestations [16]. In the present study, we focus on one specific subtype, craniotubular dysplasia, Ikeqawa type (CTDI).

CTDI is an extremely rare skeletal disorder. The number of published cases remains limited. A recent review published in December 2025 identified a total of 14 reported cases from eight unrelated families [3]. With the addition of a newly reported case published in February 2026, together with our current Bahraini case, the total number of documented CTDI cases increases to approximately 16 [12].

CTDI was first described by Guo et al. (2021), who identified pathogenic variants in the TMEM53 gene as the cause of a previously unclassified SBD. The study involved five patients from four unrelated Indian families, and genetic analysis confirmed an autosomal recessive pattern of inheritance. Two distinct TMEM53 pathogenic variants were identified: c.222_223insCATG in one family and c.62-5_62-3delITTC in the remaining three families [7]. Clinically, all five patients presented with short stature and facial dysmorphic features, including macrocephaly, dolichocephaly, prominent forehead, epicanthic folds, and thick vermilion of the lips. A key and consistent finding was progressive vision loss starting in early childhood, attributed to optic nerve compression. Notably, none of the patients exhibited dental abnormalities, intellectual disability, or spinal or chest deformities [7]. Radiologically, the condition presented with hyperostosis of the calvaria, mild platyspondyly, and broadening of the pubic and ischial bones. The long bones showed diaphyseal under-modeling and broadening, while the short tubular bones exhibited mild shortening and diaphyseal broadening. These findings are consistent with a craniotubular dysplasia, although the specific pattern did not match any previously classified skeletal disorder, highlighting the novelty of this TMEM53-related skeletal dysplasia [7].

Since then, a new article published in 2024 contributes further to the understanding of this topic, and the clinical presentations were strikingly similar to those of four Indian families, even though this case was not consanguineous. This case describes a 10-year-old American man who presented with progressive bilateral vision loss, initially with right eye blindness and later declining left eye acuity, ultimately reaching only hand-motion perception. Identified through whole-genome sequencing as compound heterozygous for a splice-site deletion and a missense variant (c.650C>T, p.Ser217Leu). MRI revealed bilateral optic canal stenosis and dilated optic nerve sheath, with fundoscopy showing optic disc pallor and edema without raised intracranial pressure. He also presented with many skeletal phenotypes, including skeletal overgrowth with macrocephaly, scoliosis, widened long bones, and vertebral abnormalities. Radiology identified generalized bony overgrowth, and a bone density scan (DXA) revealed low bone mineral density consistent with osteoporosis [1].

Moreover, another case report published in August 2024 described three siblings of Caucasian descent diagnosed with CTDI. The patients include a 21-year-old man (Patient 1), a 20-year-old woman (Patient 2), and a 13-year-old boy (Patient 3). All three individuals were born after uneventful pregnancies and presented with similar clinical features, including characteristic craniofacial dysmorphisms such as dolichocephaly, prominent forehead, biparietal narrowing, and single palmar creases. Growth retardation with short stature was observed in all three patients. Skeletal abnormalities included broad and short hands, short metatarsals and phalanges, broad and short pelvis, and hallux valgus. Radiographic findings revealed skull base sclerosis, mild flattening of the thoracolumbar vertebrae, thickened skull, sagittal craniosynostosis, narrowing of the optic canal, and cerebellar tonsillar herniation. Genetic analysis identified that all three siblings were compound heterozygous for two novel intronic/splice variants in the TMEM53 gene: c.62-23_92del and c.62-2A>G. Interestingly, all three patients exhibited cardiac anomalies, specifically persistent ductus arteriosus (PDA), which was not reported in any previously published case of CTDI. However, the author discussed the potential role of disruption of the bone morphogenetic protein (BMP)/SMAD signaling pathway in these cardiac manifestations. However, the additional presentations that these three patients exhibited were not explained to be related to CTDI or urinary tract problems, maldescended testis, and juvenile arthritis [9].

Several publications have reported an association between this syndrome and reduced bone mineral density. However, only one article published in 2024 provided detailed information regarding osteoporosis management [1]. Osteoporosis is a common disorder characterized by reduced bone strength, predisposing older individuals to an increased risk of fractures [17]. Up to half of male osteoporosis cases are attributed to secondary causes, most commonly hypogonadism, excessive alcohol consumption, and chronic glucocorticoid use [18].

In this case, zoledronic acid was started as antiresorptive therapy (1 mg every three months, later 2 mg). It strongly inhibits osteoclast-mediated bone resorption. This was justified because the patient had prior glucocorticoid use. Glucocorticoids suppress osteoblasts and prolong osteoclast survival. The treatment was well tolerated, with no infusion-related side effects. Following therapy and vitamin D, the patient’s bone mineral density Z-scores improved to low-normal range [1].

The last published article on the PubMed database on CTDI was in February 2025, reporting two Iranian siblings from a consanguineous family carrying a novel homozygous missense variant in TMEM53 (c.704G > T, p.R235L) that co-segregates with disease. They were referred for progressive, severe visual loss of initially unknown origin. Ophthalmologic evaluation revealed severe optic atrophy and optic canal narrowing, which explained their visual deterioration. Radiographic findings typical of CTDI included hyperostosis of the calvaria and skull base, undermodeling of metadiaphyses of long tubular bones, and mild shortening and broadening of diaphyses in short tubular bones. The older sibling underwent transnasal endoscopic optic canal decompression, resulting in significant improvement in visual acuity and daily visual function, underscoring the benefits of early surgical intervention [19].

Our case stands out among the limited number of reported cases of CTDI because of its distinctly atypical presentation and its comprehensive clinical, radiological, and genetic characterization. Unlike previously described cases with childhood onset - often marked by cranial dysmorphism and early vision loss - our patient presented in adulthood, with symptoms beginning almost in the third decade of life and progressing gradually over a 10-year period. Notably, the initial manifestations were spinal rather than craniofacial, consisting of progressive pain and myelopathy, thereby expanding the recognized clinical spectrum of the disease.

Detailed spinal imaging revealed extensive spinal canal stenosis and significant cord compression, particularly in the cervical region. The depth and clarity of spinal documentation in this case provide, to our knowledge, the most comprehensive imaging description of spinal involvement in CTDI to date. This emphasizes the importance of thorough spinal assessment in patients with suspected or confirmed CTDI, even in the absence of prominent early cranial features.

Genetically, this case is equally remarkable. We identified two novel compound heterozygous TMEM53 variants - p.Ala145Pro and p.Pro211Thr - that have not been previously reported. Although segregation analysis could not be performed due to the unavailability of parental samples, the patient’s clinical and radiological findings were highly consistent with the phenotype of TMEM53-related CTDI, strongly supporting the pathogenic relevance of these variants. This report, therefore, expands the mutational and allelic diversity of TMEM53 and emphasizes that a diagnosis can be confidently proposed even without a confirmed inheritance phase when the phenotype-genotype correlation is compelling.

## Conclusions

This case describes a rare adult-onset presentation of CTDI, caused by a novel compound heterozygous TMEM53 variant. The predominance of spinal manifestations, accompanied by extensive spinal imaging findings, makes this report a particularly informative and meaningful contribution to the existing CTDI literature.

This case further underscores the importance of considering CTDI in adults presenting with unexplained spinal pathology and visual impairment. It highlights the critical role of comprehensive genetic evaluation, particularly whole-exome sequencing, in patients with atypical skeletal disorders. Moreover, it emphasizes the need for continued research to better delineate the natural history, refine management strategies, and clarify the long-term outcomes of TMEM53-related skeletal dysplasia.
